# Supplementary material for: A Systematic Review of Genetic Polymorphisms Associated with Bipolar Disorder Comorbid to Substance Abuse
Source: Genes (Basel). 2022 Jul 22;13(8):1303. doi: 10.3390/genes13081303 (PMC9330731; doi:10.3390/genes13081303)
Supplement: Supplementary file 1 [file genes-13-01303-s001.zip › genes-1827503-supplementary/Table S1/NOS.docx.html]

 

|  |  |  |  |  |  |  |  |  |  |
| --- | --- | --- | --- | --- | --- | --- | --- | --- | --- |
|  | Selection | | | | Comparability | Exposure | | |  |
| Article | 1 | 2 | 3 | 4 | 1 | 1 | 2 | 3 | Tot ☆ |
| Banach et al. (2018) | a ☆ | a ☆ | a ☆ | a ☆ | a ☆ (age)  b ☆ (sex) | a ☆ | a ☆ | b | 8 |
| Bortolasci et al. (2014) | a ☆ | a ☆ | a ☆ | a ☆ | a ☆ (age) | a ☆ | a ☆ | b | 7 |
| Bortolasci et al. (2014a) | a ☆ | a ☆ | a ☆ | a ☆ | a ☆ (age) | a ☆ | a ☆ | b | 7 |
| Chang et al. (2015) | a ☆ | a ☆ | a ☆ | a ☆ | a ☆ (age) | a ☆ | a ☆ | b | 7 |
| Cui et al. (2011) | a ☆ | a ☆ | a ☆ | a ☆ | a ☆ (age)  b ☆ (sex) | a ☆ | a ☆ | b | 8 |
| Gorwood et al. (2000) | a ☆ | a ☆ | a ☆ | a ☆ | a ☆ (age)  b ☆ (sex) | a ☆ | a ☆ | b | 8 |
| Gratacòs et al. (2008) | a ☆ | a ☆ | a ☆ | a ☆ | a ☆ (age)  b ☆ (sex) | a ☆ | a ☆ | b | 8 |
| Hartz et al. (2011) | a ☆ | a ☆ | c | a ☆ | a ☆ (age)  b ☆ (sex) | a ☆ | a ☆ | b | 7 |
| Huang et al. (2003) | a ☆ | a ☆ | a ☆ | a ☆ | a ☆ (age) | a ☆ | a ☆ | b | 7 |
| Lydall et al. (2011) | a ☆ | a ☆ | a ☆ | a ☆ | a ☆ (age)  b ☆ (sex) | a ☆ | a ☆ | b | 8 |
| Mazza et al. (2010) | a ☆ | a ☆ | a ☆ | a ☆ | a ☆ (age) | a ☆ | a ☆ | b | 7 |
| Mandelli et al. (2011) | a ☆ | a ☆ | a ☆ | a ☆ | a ☆ (age) | a ☆ | a ☆ | b | 7 |
| Novak et al. (2010) | a ☆ | a ☆ | a ☆ | a ☆ | a ☆ (age)  b ☆ (sex) | a ☆ | a ☆ | b | 8 |
| Prossin et al. (2018) | a ☆ | a ☆ | a ☆ | a ☆ | a ☆ (age) | a ☆ | a ☆ | b | 7 |
| Reginsson et al. (2017) | a ☆ | a ☆ | a ☆ | b | a ☆ (age)  b ☆ (sex) | a ☆ | a ☆ | b | 7 |
| Sharp et al. (2014) | a ☆ | a ☆ | a ☆ | a ☆ |  | a ☆ | a ☆ | b | 6 |
| Szczepankiewicz et al. (2006) | a ☆ | a ☆ | a ☆ | a ☆ | Age ☆  b ☆ (sex) | a ☆ | a ☆ | b | 8 |
| Mean | 7,35 | | | | | | | | |
